# Armored Geomembrane Cover Engineering

**DOI:** 10.3390/ijerph8062240

**Published:** 2011-06-16

**Authors:** Kevin Foye

**Affiliations:** CTI and Associates, Inc., 51331 W. Pontiac Trail, Wixom, MI 48393, USA; E-Mail: kfoye@cticompanies.com; Tel.: +1-248-486-5100; Fax: +1-248-486-5050

**Keywords:** environmental engineering, geomembranes, geocells, water contamination

## Abstract

Geomembranes are an important component of modern engineered barriers to prevent the infiltration of stormwater and runoff into contaminated soil and rock as well as waste containment facilities—a function generally described as a geomembrane cover. This paper presents a case history involving a novel implementation of a geomembrane cover system. Due to this novelty, the design engineers needed to assemble from disparate sources the design criteria for the engineering of the cover. This paper discusses the design methodologies assembled by the engineering team. This information will aid engineers designing similar cover systems as well as environmental and public health professionals selecting site improvements that involve infiltration barriers.

## 1. Introduction

Geomembranes are a class of geosynthetic (a plastic used in conjunction with earthwork construction) that act as barriers to the movement of water and other liquids. The most common geosynthetic products used to restrict water infiltration are geomembranes and geosynthetic clay liners (GCLs). When properly installed and protected, geomembranes and GCLs offer an effectively impervious barrier to water. One prominent application of geomembranes is as part of a cover system. Cover systems are a common feature in waste containment practice. These systems are used to prevent the infiltration of surface or storm water into wastes or other subsurface materials, typically because such infiltration would result in the transport of contaminants via the infiltrated water.

Geosynthetic cover systems as traditionally implemented in North American waste containment practice involve the installation of geomembranes and/or GCLs beneath soil cover systems. The soil cover system serves in numerous roles, including infiltration barrier, erosion control, geomembrane armor, geomembrane ballast, and vegetation support. Vegetation is often needed to limit erosion of the soil cover by precipitation. Most of these cover systems achieve overall stability through the frictional resistance of the cover to sliding down the slope. When such sliding does occur, it generally is along the interfaces between the different layers of the cover slope [1].

**Figure 1.**
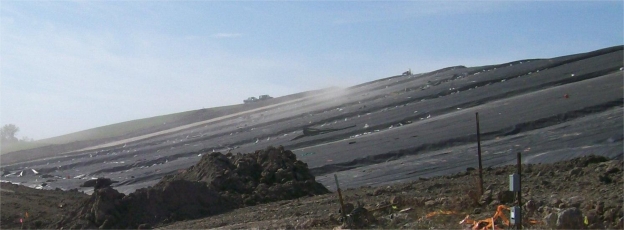
Photo of 1.0-mm thick HDPE exposed geomembrane cover. Soil windrows capped with geomembrane are visible in shadowed relief on the slope face.

Exposed geomembranes, such as the one shown in Figure 1, undoubtedly offer benefits such as lower construction and maintenance costs compared to geomembrane barriers overlain by soil cover. Exposed geomembrane covers are especially attractive for temporary or interim (e.g., less than 20 years) cover applications [2]. However, exposed geomembranes are susceptible to dislocation by a number of forces. Chief amongst these forces is wind uplift.

Figure 1 also shows typical soil windrows used to ballast the exposed geomembrane against wind uplift. These specific windrows were sized to resist uplift through friction and gravity. The wind loading placed on the system was calculated using the methodology of Giroud *et al.* [3] while the load capacity and stability of the windrows was analyzed using a sliding block analysis. Erosion of the soil windrows is prevented by a geomembrane cap welded over the ballasting soil. The windrows are held in place by friction with the underlying textured geomembrane. The ballasting system described above is sufficient for slopes shallow enough (e.g., 1 vertical: 3 horizontal) to support the cover system through friction. Steeper slopes require a support system similar to the one described in the following case history.

The geomembrane component of an exposed geomembrane cover system is susceptible to damage from a variety of sources. Example sources include falling debris, blown debris, burrowing animals, human vandalism, and motorized vehicles. Fortunately, the risk of damage to steep exposed geomembrane systems from motorized vehicles is limited by the inability of most vehicles to climb steep slopes. However, the remaining issues must be addressed. Falling debris is a major concern for cover systems installed partly across the face of natural slopes where rocks, trees, and other debris may fall onto the slope face. Falling debris is also a concern for slope covers installed below roadways, industrial facilities, or other inhabited areas. Risk of damage from these sources can be mitigated through the selective use of small embankments and other debris barriers. Risk of damage from vandalism can be reduced by restricting access through fences—a typical precaution in waste containment facilities.

In the case of slopes where it is impractical to control the incidence of debris or other types of damage to the cover system, the engineer has the option to design an armor system to protect the geomembrane. The following case history describes such an armor system. The armor system includes a 100-mm layer of crushed stone aggregate. In this case history, the decision to use limestone aggregate as the armor media was influenced by aesthetic concerns, project economics, armor efficacy, practical limitations of alternate media, and material availability.

In order to successfully design and construct a steep geomembrane cover system similar to the one described in this paper, a number of technical issues must be addressed. Each of these issues is well within the capability of geotechnical engineers to address. However, the project team’s experience with steep slope barrier installations suggests that some further background information will be useful to practitioners contemplating the use of these systems. The following sections discuss a number of these specific technical issues that engineers must resolve in the design and construction of steep slope barrier systems.

## 2. Case History Background

### 2.1. Project History

This paper describes the 2006 to 2009 design and construction of a geomembrane cover to prevent stormwater infiltration to pyrite-bearing rock along part of the newly constructed Interstate-99 (I-99) corridor in central Pennsylvania. The I-99/State Route 6220 Project extends from the Village of Bald Eagle in Blair County, PA to the Mount Nittany Expressway (U.S. Route 322) in Centre County, PA. The project involved the construction of a four-lane limited access highway with four interchanges and approximately 29 kilometers of roadway. The project is part of a much larger transportation project to extend I-99 to I-80. Section 12 of the project extends from North Bald Eagle Creek to the Mount Nittany Expressway. This section includes the area of concern regarding the pyrite-bearing rock.

Construction of Section 12 involved one major rock cut and several smaller rock cuts. Material from the rock cut was subsequently used to construct bridge abutments, highway embankments, and other earth fill applications. In one application, a nearly 1.6-kilometer long segment of highway was bifurcated to allow the buttressing of a sliding rock slope. Therefore, rock removed from the large cut became integral to the structure of several highway features.

**Figure 2.**
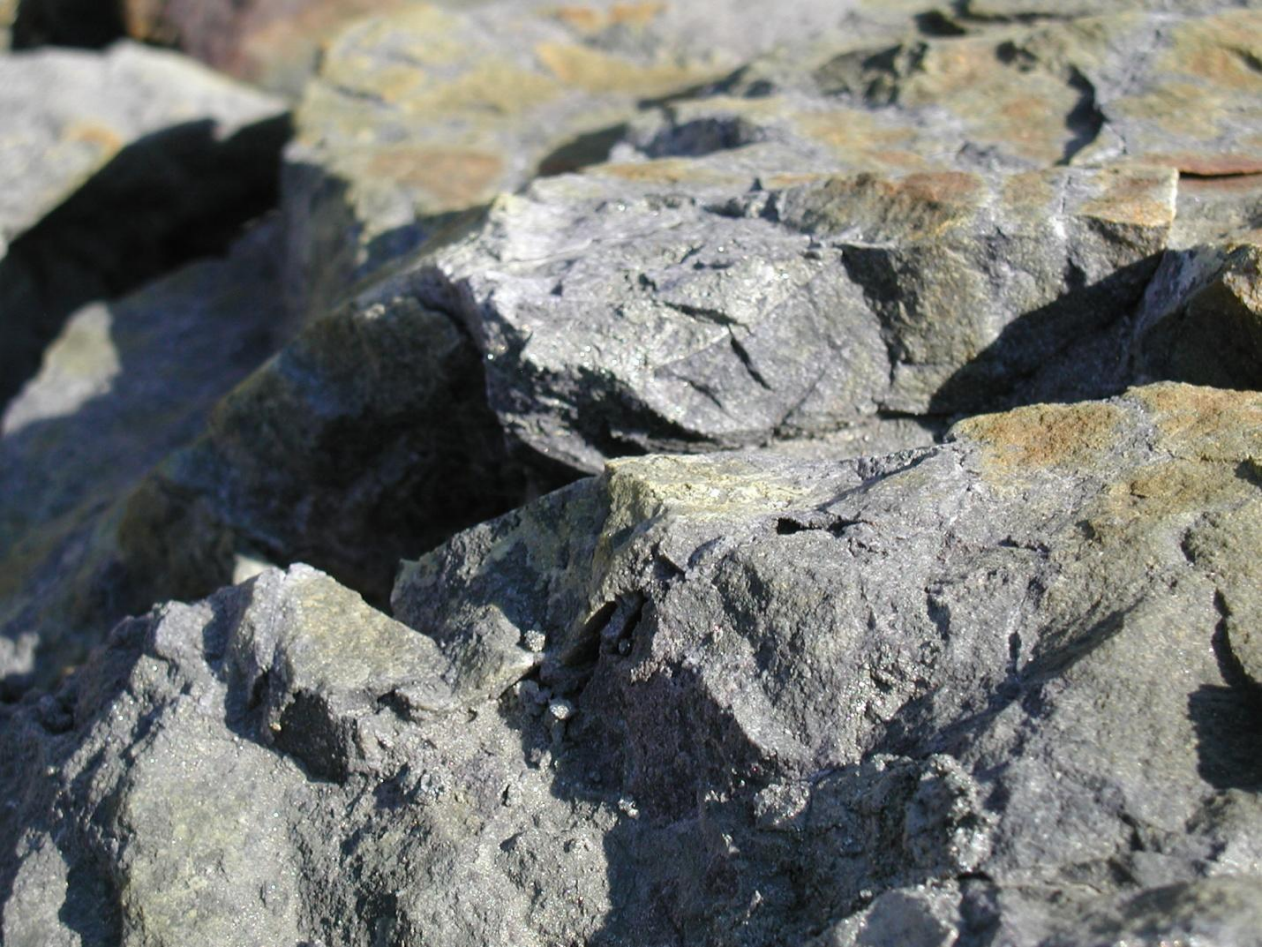
Photo showing exposed pyrite. Pyrite deposits appear as lighter-colored streaks compared to the surrounding rock.

During construction of Section 12 in 2003, pyrite-bearing sandstone was exposed to air and precipitation, creating Acid Rock Drainage (ARD) containing elevated concentrations of heavy metals and sulfates. A photo showing the typical appearance of pyrite during the project is shown in Figure 2. The acidity is caused by the reaction of sulphide minerals with oxygen and water. The acid dissolves and leaches minerals from the rock, further degrading water quality. The runoff from these areas threatened the quality of two adjacent exceptional value trout streams and local residential water wells.

Within Section 12, eleven areas containing pyrite-bearing rock were identified. These areas included the rock cuts supplying this rock, temporary stockpiles of excavated rock, and permanent embankments constructed from this rock. The pyrite-bearing rock in many of the temporary stockpiles could be safely removed and transported to a disposal site for permanent encapsulation. Project managers referred to these areas as movable areas. Other areas, such as the original rock cuts, a constructed rock buttress stabilizing a cut slope, and embankments beneath constructed bridge abutments were considered immovable. The pyrite-bearing rock in these immovable areas could not be moved without impairing human safety or incurring unacceptable costs.

The Pennsylvania Department of Transportation (PennDOT) entertained several design concepts to mitigate the exposed pyrite in the immovable areas. The objective of the proposals was to encapsulate the pyrite-bearing rock to prevent Acid Rock Drainage (ARD) from becoming stormwater runoff.

A search for a remediation plan delayed the project three years. Following a series of meetings between PennDOT, the Pennsylvania Department of Environmental Protection (PADEP), and the host community, all parties agreed to a remediation program consisting of two different solutions to encapsulate the movable and immovable rock. The estimated 1,000,000 cubic meters of movable rock was disposed in a double-lined landfill constructed along the right-of-way of the project. Landfill construction and rock placement was sequenced to minimize stormwater runoff.

For the immovable material, the parties agreed a conceptual design to construct a geomembrane cover over the exposed rock slopes. PennDOT gave approval to begin a full design and preparation of a permit application. Both the permit and design was subject to the review and approval of PennDOT and PADEP. Thus, the selected solution for the approximately 140,000 square meters of exposed rock slopes encompassing the immovable material area was to construct a geomembrane cover. The case history presented in this paper discusses the engineering of the immovable rock slope cover system.

### 2.2. Cover Design

**Figure 3.**
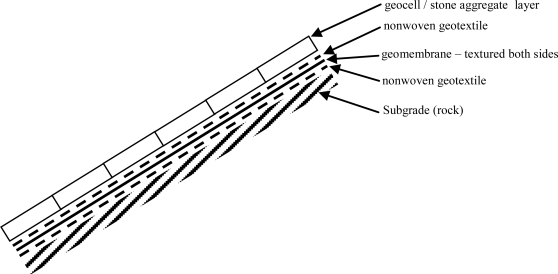
Slope protection system design cross section.

The cover consists of a High Density Polyethylene (HDPE) Geomembrane protected by two nonwoven geotextiles and a crushed stone-filled geosynthetic cellular confinement system (geocell) layer as shown in Figure 3. Due to the steepness of the slopes to be covered—most slope inclinations were steeper than 3 horizontal-to-1 vertical (3H:1V)—a support system to transfer the dead load of the stone-filled geocells to the crest of slope was needed. While the use of geocells for erosion control is not novel, project-specific requirements created two interesting design challenges: (1) no penetrations through the geomembrane were allowed on the slope face and (2) limited upslope area was available for anchor construction. These constraints demanded significant engineering consideration to arrive at an economical design for each section.

**Figure 4.**
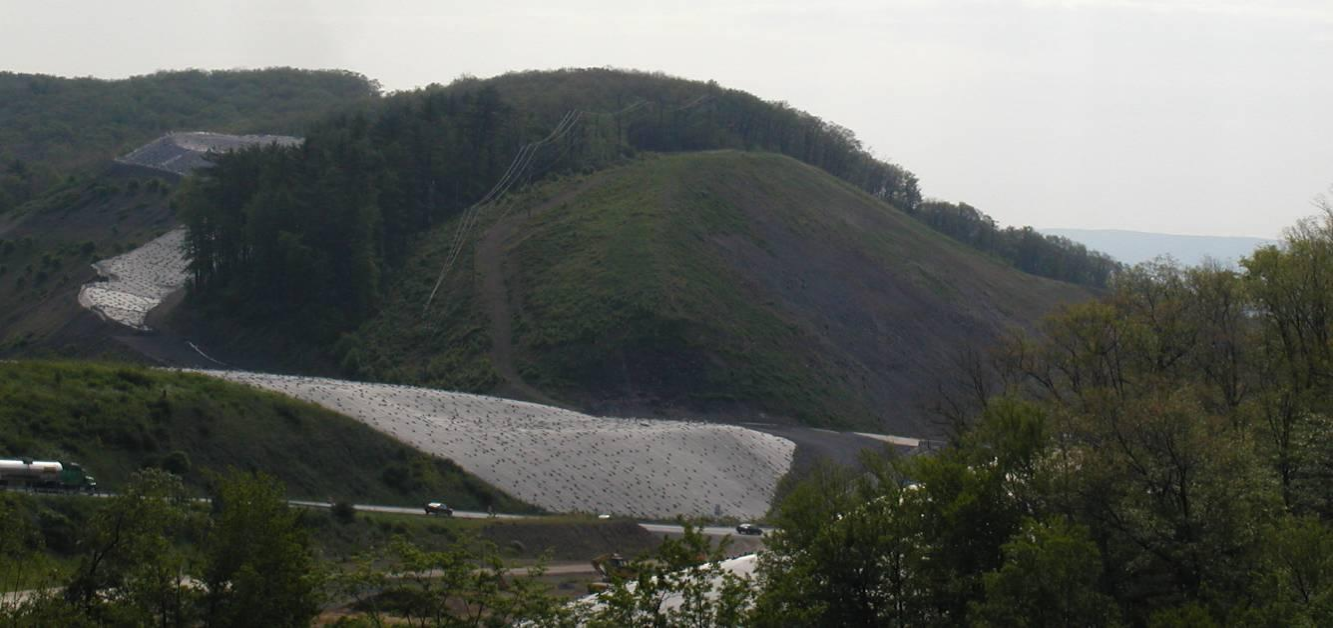
Photo of the Large Cut Face and neighboring temporary stockpiles (covered in black PVC geomembrane) prior to cover system construction. Vehicles are shown for scale.

The immovable rock slopes range in length from 9.0 meters to 110 meters with slope inclinations varying from 3H:1V to 1H:1V. The most severe slope—130 meters long, 1.5H: 1V—known on-site as the Large Cut Face, is shown in Figure 4. Due to the various lengths and slope inclinations, a general slope cover system design was adapted to meet the specific demands of each section. Specifically, the slope length strongly influenced the reinforcement demand while the topography of the crest of slope limited the range of practical anchor types and dimensions.

The geosynthetic cover design cross section is presented in Figure 3. The cover system consists of four layers: (1) a 540 g/m^2^ nonwoven geotextile bottom cushion, (2) a 1.0 mm HDPE geomembrane, (3) a 540 g/m^2^ geotextile top cushion, and (4) a 100-mm deep, stone-filled geocell. The top and bottom cushions were selected to protect the geomembrane against damage by the crushed stone selected for the protective cover and irregularities in the subgrade, respectively. The adequacy of the 540 g/m^2^ geotextile cushion layers was evaluated according to the methodology of Wilson-Fahmy *et al.* [4] by considering a range of possible protrusion heights under the action of construction foot traffic as well as the weight of the stone armor layer. The HDPE geomembrane acts as the infiltration barrrier. The crushed stone aggregate-filled geocell layer armors the geomembrane against damage.

## 3. Armor Support

Including an armor layer as part of a steep slope cover system poses an additional support concern. Adequate support must be engineered to prevent the armor layer from sliding off the slope. Conditions favorable for sliding failures are exacerbated by the presence of the geomembrane, which reduces the available friction to resist sliding [1]. In the case of traditional waste containment cover systems with slope inclination less than 14 degrees (shallower than 3H: 1V), the friction between the various soil and geosynthetic layers is typically adequate to resist sliding, such as in Figure 1, despite this reduction in friction. However, for steep cover systems, friction alone is often insufficient to support the weight of the armor layer. In these cases, additional structural support is required. Furthermore, in the case of the selected stone aggregate armor system, erosion of the armor layer is also a major concern. In this case history, erosion control is provided by geocells embedded within the stone.

Because friction alone was insufficient to prevent sliding of the cover system, the cover armor required additional support from tension reinforcement members anchored at the crest of slope. Two different reinforcement techniques were applied: (1) geogrid reinforcement beneath the geocell layer and (2) stainless steel wire rope reinforcement of the geocell.

**Figure 5.**
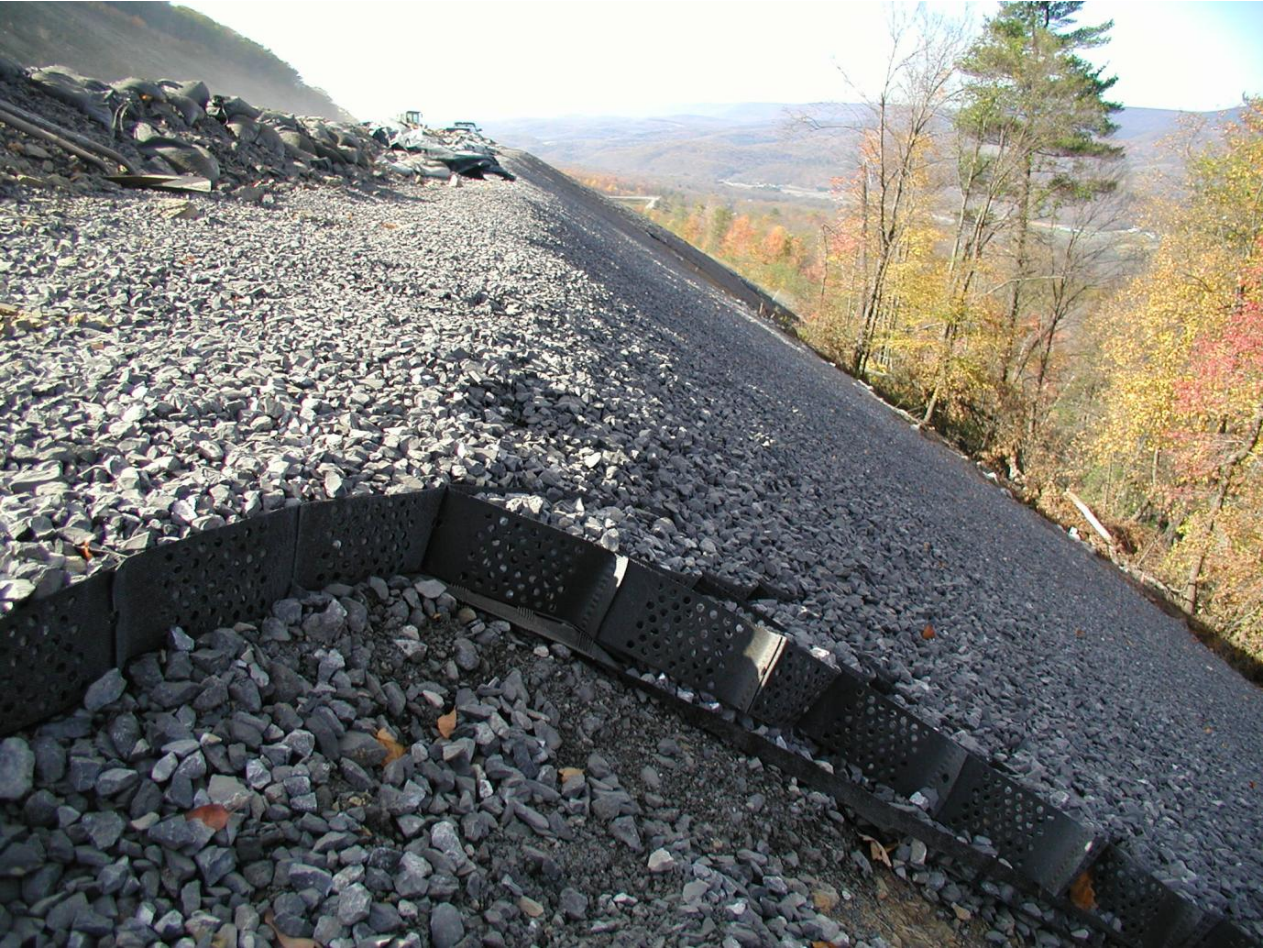
Stone aggregate-filled geocell overlying a geogrid layer. The slope shown varies in inclination from 1H:1V to 1.5H:1V.

In the case of geogrid reinforcement, interlock of the stone aggregate with the geogrid provided the needed load transfer from armor to the reinforcement. Figure 5 shows an example section where the aggregate interlock with the geogrid is clearly visible. The geogrid itself is anchored through friction beneath soil ballast at the crest of slope.

In the case of very long slopes or slope crests with limited room for anchorage, steel wire rope, networked together with the geocells was used as the tension members in lieu of geogrid. The network of geocells and steel tendons provides the load transfer from the armor system to the reinforcement. Load transfer from the tendons to the ground was accomplished by anchors at the slope crest. Armor system anchorage is discussed in section 4.

### 3.1. Geogrid

When geogrid is used to reinforce the cover system, the geocell only provides lateral confinement and erosion control, with all structural load being transmitted directly from the cover material to the geogrid. The following subsections describe the engineering calculations to select the proper geogrid.

#### 3.1.1. Interface Friction

The first step in the analysis of the slope protection system is an evaluation of the frictional properties of the interfaces created by the proposed design. Interfaces exist when dissimilar materials come in contact. The frictional properties of these interfaces depend on test conditions including normal loading, rate of shearing, degree of submergence, size of apparatus, *etc.* [5]. Table 1 tabulates the interface shear parameters for these sideslope system interfaces. Values listed in Table 1 were selected based on representative direct shear test databases reported by Koerner and Narejo [6].

After examining all of the interfaces listed in Table 1, the critical interface was identified as that between the nonwoven geotextile and the textured geomembrane, with an interface friction angle of 26°. Direct shear testing of this interface was performed to determine the actual value for final design. Direct shear testing of the specific products used in construction yielded a design interface friction angle of 22°for the critical interface.

Applying sliding-block (“infinite slope”) type analysis, the factor of safety against sliding resisted only by friction is

(1) $$FS=\frac{N\;\text{tan}\;\delta }{F_{D}}=\frac{W\text{cos}\;\beta \text{tan}\delta }{W\text{sin}\beta }=\frac{\text{tan}\delta }{\text{tan}\beta }$$

where *N* is the force normal to the slope face, *δ* is the interface friction angle (22°), *F_D_* is the force tangential to the slope (the driving force, directed downslope), *W* is the weight of the cover system, and *β* is the slope inclination. For the project’s typical slope inclination of 1.5H:1V (33.7°), the resulting factor of safety is 0.61, indicating that interface friction alone is insufficient to resist sliding.

#### 3.1.2. Geogrid Reinforcement

The use of geogrid reinforcement to support cover systems has been previously addressed by Koerner and Soong [1]. Load transfer from the armor system to the geogrid reinforcement is via interlock of the stone aggregate infill with the geogrid (visible in Figure 5). Forces driving sliding include the self weight of the armor and any overburden (e.g., snow). Forces resisting sliding include interface friction and tension in the geogrid. Engineering of the geogrid primarily concerns the selection of a geogrid with sufficient long-term strength to prevent sliding. The required reinforcing geogrid strength was determined following design procedures adapted from Koerner [5], with the exception that passive resistance from the toe of slope is ignored since such toe contact is not available for several slopes on-site. Design conditions for the sizing of the geogrid reinforcement were based on scenarios established through coordination between PADEP and the design engineer. The basic design parameters for the two principal design scenarios are summarized in Table 2.

Introducing to Equation 1 the allowable resisting force from the geogrid for a slope of finite length yields the following equation for the factor of safety:

(2) $$FS=\frac{N\text{tan}\delta +T_{all}}{F_{D}}=\frac{W\text{cos}\beta \text{tan}\delta +T_{all}}{W\text{sin}\beta }=\frac{L(d\gamma_{i}+q)\text{cos}\;\;\beta \text{tan}\delta +T_{all}}{L(d\gamma_{i}+q)\text{sin}\beta }$$

where *T_all_* is the geogrid allowable tension, *L* is the slope length, *d* is the geocell depth, *γ_i_* is the infill unit weight, and *q* is the overburden force per unit area. Using a target factor of safety = 1.5, the allowable tension in the geogrid is 32.9 kN/m and 34.0 kN/m for the snow and ice design scenarios, respectively, for a 20-m long slope at a 1.5H:1V inclination. This slope and inclination is representative of one area of the cover reinforced with a geogrid. Based on the calculated allowable tension, the ice design scenario controls the specification of geogrid strength. The calculated allowable tension was further factored by the recommended reduction factors for installation and long-term effects to obtain the design strength:

(3) $$T_{d}=RF\times T_{all}$$

where *T_d_* is the design geogrid tensile strength and *RF* is the cumulative reduction factor (4.0) accounting for installation damage, creep, and chemical degradation (value adapted from recommendations by [5]). This design value *T_d_* is the design ultimate strength specified for the geogrid for the example design slope. For the 20-m slope example given above, the minimum design ultimate tensile strength specified for the geogrid was 136 kN/m. This value was verified by wide-width tensile testing of the selected geogrid product. This design procedure was repeated for all slopes selected to receive geogrid reinforcement. Throughout the entire cover system design, specified ultimate tensile strengths were standardized to three representative values to simplify construction logistics and avoid misplaced geogrids.

### 3.2. Steel Tendons

Several slopes were too long to reinforce with geogrids for the following practical reasons: (1) commonly available geogrids were not strong enough (*T_d_* > 220 kN/m), and/or (2) insufficient space was available at the crest of slope for an economical gravity anchor. For these slopes, stainless steel wire rope tendons were designed to provide the required reinforcement.

**Figure 6.**
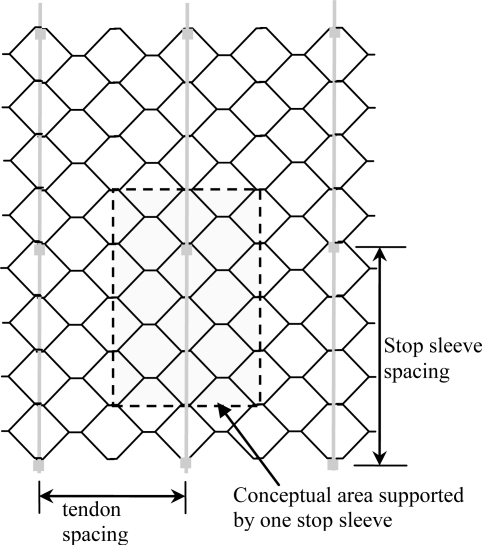
Key tendon design parameters for geocell reinforcement.

When geogrid is used to reinforce the cover system, the geocell only provides lateral confinement and erosion control, with all structural load being transmitted directly from the cover material to the geogrid. When the stainless steel tendons are used, the geocell also functions as a load path from the cover material to the reinforcement. Load from the geocell infill is transferred to tendons through tension in the geocells and then stop sleeves spaced at regular intervals within each geocell panel. The key features of the tendon design are tendon strength, tendon spacing, and stop sleeve spacing (Figure 6). The same driving forces discussed for geogrid reinforcement applied to the steel tendon design. The basic design parameters for the two principal design scenarios are summarized in Table 2.

#### 3.2.1. Stop Sleeve Spacing

**Figure 7.**
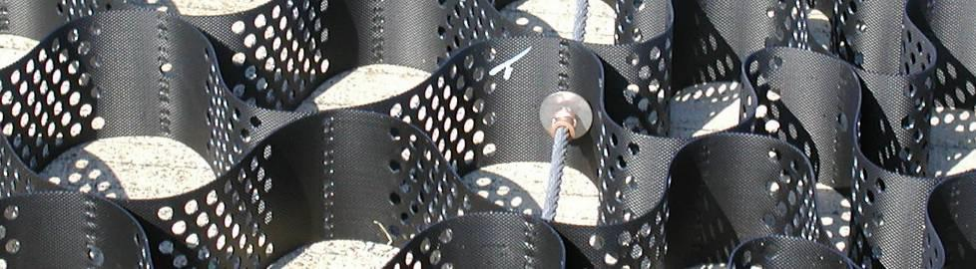
Photo showing typical installed copper stop sleeve/stainless steel washer bearing assembly inside an empty geocell.

A key consideration in the reinforcement application of geocells is the amount of load that can be effectively transmitted from the tendon to the geocell. Load is transmitted from the geocell to the tendon through a bearing washer and stop sleeve assembly (Figure 7).

The connection of the stop sleeve to the tendon is well understood in rigging practice (e.g., for lifts with cranes). However, the bearing resistance of the washer against the geocell is less understood. Therefore, a laboratory study of this pullout resistance was undertaken by the Geosynthetic Research Institute (GRI). In this study, three different types of tests were performed on the geocells: (1) a wide width tension test of the geocell wall (a test similar to ASTM D4885 [7]), (2) a seam peal test to evaluate the junction strength (a test similar to ASTM D6392 [8]), and (3) a pullout test simulating the pullout of a stop sleeve/washer assembling from the geocell panel. Table 3 summarizes the average test values obtained from replicate tests.

**Figure 8.**
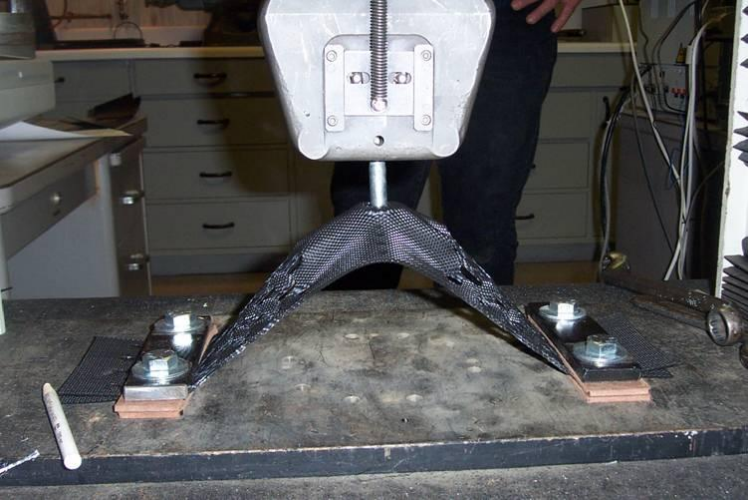
Photo of tendon connection pullout test.

**Figure 9.**
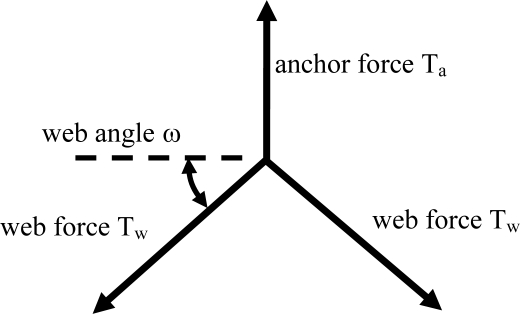
Free body diagram of forces acting on geocell washer/stop sleeve bearing assembly.

For the pullout tests, a prototype washer assembly was pulled through 2-ply geocell wall to simulate the loading in the field (Figure 8). The maximum connection load between the geocell and tendon has three possible limits: (1) the bearing strength of the cell wall behind the washer, (2) the tensile strength of the geocell wall, and (3) the junction strength of the geocell. The ultimate load obtained from the GRI pullout test was 2,890 N for the specified 100-mm deep, 1.3-mm thick HDPE geocell. This value is exactly twice the value predicted by the equilibrium of forces in free body diagram of the single-ply tendon/geocell connection shown in Figure 9. With a web angle *ω* = 45°and a web force *T_w_* = 1,030 N, the anchor force *T_a_* = 1,460 N (about half of the simulated pullout test result). Thus, the pullout strength for these connections is limited by the tensile strength of the adjacent perforated geocell wall sections. This conclusion is supported by the failure mode observed in the laboratory. Washers installed in production geocell panels bear against welded cell junctions where the wall material is double in thickness and upslope armor is partially supported in compression. Therefore, a further margin of safety is provided that is not modeled using this test.

Based on the results of the GRI laboratory study, engineering calculations were performed to determine the maximum allowable stop sleeve spacing for the proposed geocell system. The maximum spacing between stop sleeve/washer assemblies depends on the available strength of tendon/geocell connections and the driving forces within the geocell armor system. Adapting Equation 2, the factor of safety against pullout of a single stop sleeve/washer assembly can be calculated as:

(4) $$FS=\frac{Lw(d\gamma_{i}+q)\text{cos}\beta \text{tan}\delta +T_{a}}{Lw(d\gamma_{i}+q)\text{sin}\beta }$$

where *w* is a representative width of the geocell layer. Considering a target *FS* = 1.5 and the controlling ice design scenario (*δ* = 0, *q* = 0) from Table 2, the maximum area *A_max_* of the geocell panel that can be supported by a single single stop sleeve/washer assembly is:

(5) $$A_{\text{max}}=Lw=\frac{T_{a}}{FS\;d\gamma_{i}\text{sin}\beta }$$

In the case of the project’s typical 1.5H:1V slope, Equation 5 yields a maximum support area A_max_ = 0.86 m^2^. Therefore, for a typical geocell panel measuring 2,550 mm wide by 8,323 mm long (21.2 m^2^ panel area), a minimum of 25 stop sleeve/washer assemblies are required. In the case of the project’s steepest slopes, 1H:1V inclination, Equation 5 yields a maximum support area A_max_ = 0.68 m^2^, requiring a minimum of 32 stop sleeve/washer assemblies per panel. The minimum 32 assemblies/panel specification was adopted for all slopes to standardize construction details. Typical design details for the project distributed these assemblies evenly over the geocell panel area.

#### 3.2.2. Tendon Spacing

The required tendon spacing depends on the length of slope supported by the tendon, the inclination of the slope, the strength of the tendon, and the frictional resistance offered by the geosynthetic interface. Similarly to the analysis of geogrid reinforcement, the stability of tendon-reinforced slopes can be analyzed using sliding block analysis. Following Equation 4, the factor of safety against sliding is computed:

(6) $$FS=\frac{Lw(d\gamma_{i}+q)\text{cos}\;\;\beta \;\text{tan}\;\delta +nT_{ult}}{Lw(d\gamma_{i}+q)\text{sin}\beta }$$

where *n* is the number of tendons per panel width *w* and *T_ult_* is the ultimate breaking strength of each tendon. The minimum tendon breaking strength specified for the project was *T_ult_* = 57.8 kN. In the case of a 105-m long 1.5H:1V slope analyzed under the ice design scenario, a minimum of *n* = 8 tendons is required to achieve a minimum factor of safety FS = 1.50.

A discussion of appropriate factors of safety is useful to understand the design engineering philosophy for the project. Typical applications of the stainless steel wire rope product selected for the reinforcing tendons include lifting and other rigging. This use is characterized by dynamic loads, multiple load cycles, limited redundancy, mechanical wear, aggressive chemical environments (e.g., saltwater), and direct threats to human safety from overhead loads. Consequently, typical factors of safety for designs incorporating wire rope are greater than or equal to five. Note that none of these conditions is applicable to the geocell reinforcement proposed for this project, except a potential threat to human safety from falling cover system materials. The static nature of the cover system support eliminates the dynamic load and mechanical wear concerns while multiple tendons and the frictional resistance from the cover system increase the system redundancy. The design scenarios presented in Table 2 were expected to represent extreme, short-duration loading events. Nominal loading scenarios are expected to yield greater factors of safety. For example, Equation 6 yields a minimum factor of safety FS = 2.92 for a 105-m long 1.5H:1V slope when using the measured values of *T_ult_* 77.8 kN, *δ* = 22°, *γ_i_* = 18.1 kN/m^3^, and *q* = 0. This condition represents the nominal design state for most of the year. The target value *FS* = 1.50 was considered appropriate for the extreme loading events.

**Figure 10.**
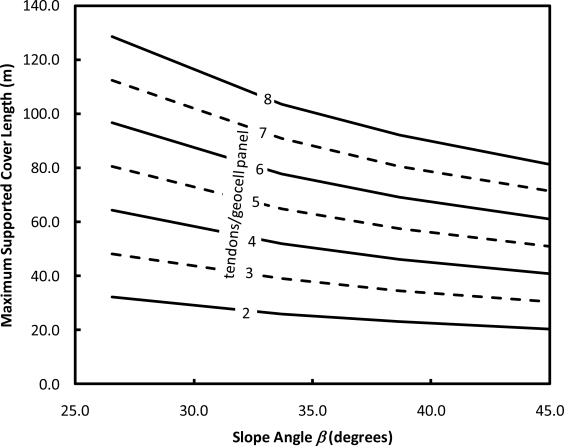
Example design chart showing maximum slope length for different slope inclinations and number of tendons per 2.55-m wide geocell panel.

The above calculations were repeated for a range of representative design slope lengths and inclinations to produce design charts used to specify the reinforcement configuration for different slopes across the site. Figure 10 presents one of these charts. The design guidance presented in Figure 10 was similarly produced in table form (Table 4) to aid the layout and quality assurance verification of different reinforcement sections.

#### 3.2.3. Elongation

**Figure 11.**
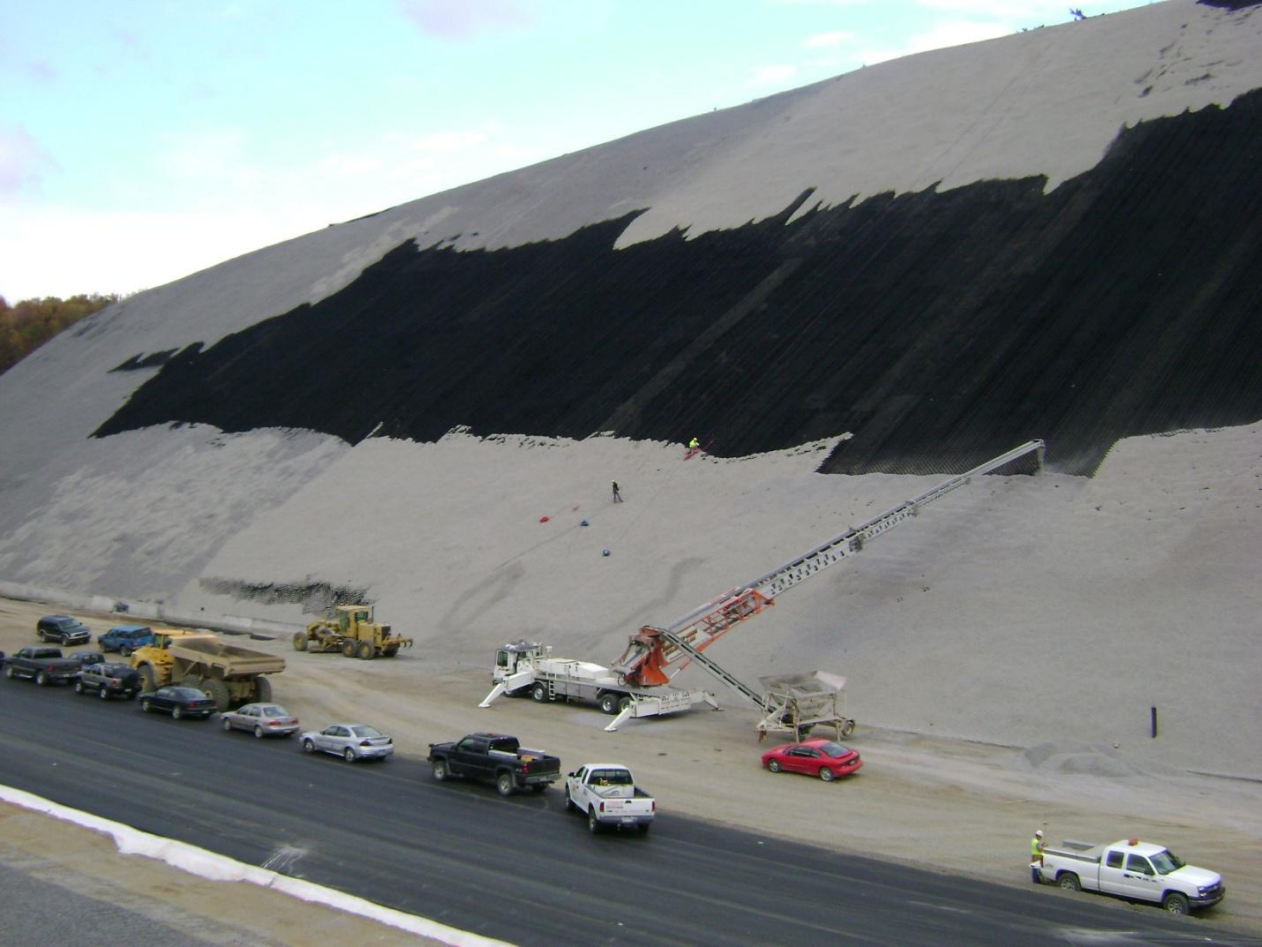
Photo showing stone aggregate infill in progress on the Large Cut Face.

An important consideration in the installation of this type of armor system is the strain compatibility between the geosythetics and the armor support system. In the case of wire rope tendons, significantly greater elongation of the tendons is required to mobilize full load than to mobilize friction in the geosynthetic layers. Therefore, the geosynthetics must be installed and anchored to tolerate strain while the armor system in being loaded. The sequence of stone infill is important in this regard. Much of the aggregate was placed in the downslope portion of the armor system first to remove slack from the tendons prior to placing stone upslope. This step was taken to minimize the required sliding of the loaded armor system on top of the geomembrane. Figure 11 presents a photo of stone infill in progress on the Large Cut Face. This photo shows crews placing stone aggregate into the geocells on the slope face with the aid of a stone slinger. Workers on the face of the slope raked the stone aggregate evenly into geocells as it was distributed by the slinger. Construction quality assurance inspectors on the project noted that infill of the bottom 1 to 2 m of the slope armor system was sufficient to remove the slack from the deployed tendons. Additional stone aggregate was required to tension and elongate the tendons.

**Figure 12.**
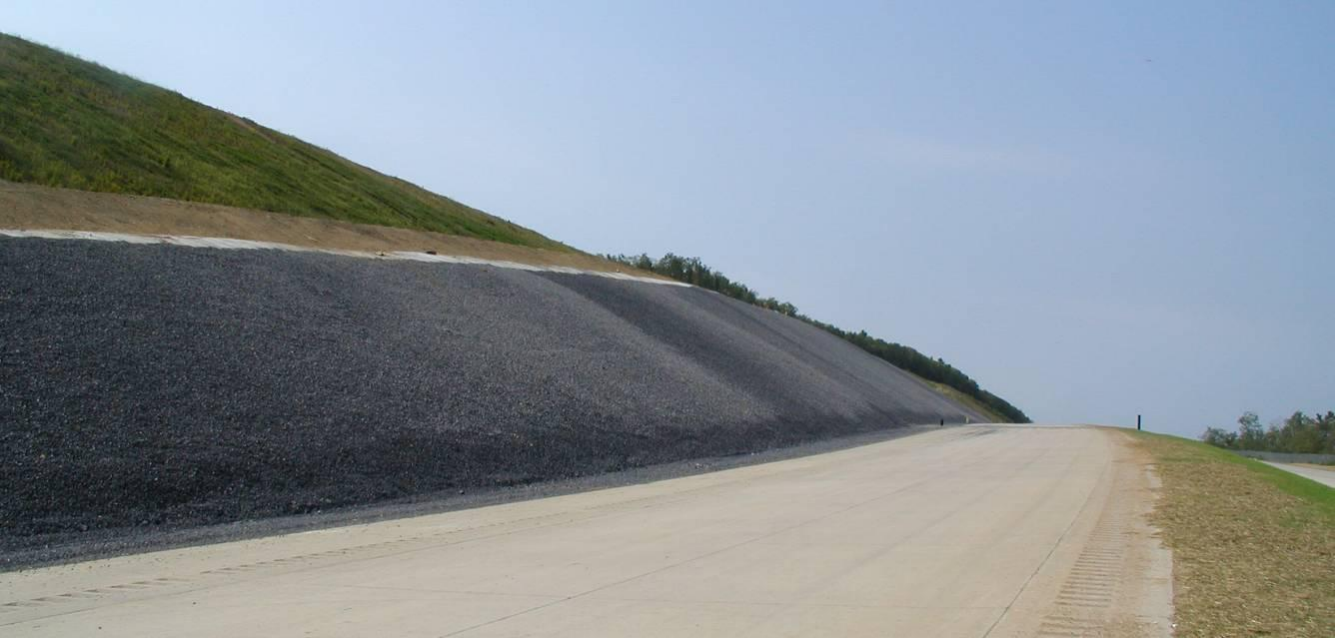
Photo showing appearance of a section of armored slope cover following aggregate infill.

The armor system design considered the tendon reinforcement as the primary support to the cover system, with the frictional resistance acting in reserve. However, the displacements required to mobilize the friction within the liner system are considerably less than those required to straighten and tension the 9 × 9 strand wire rope. Field inspection of the tendons during loading of the geocell confirmed this statement, as the tendons did not straighten in many cases until loading was nearly complete, indicating relatively minor contributions from the tendons to the load support. A photo of a completed cover system with full stone geocell infill is shown in Figure 12.

## 4. Armor System Anchorage

**Figure 13.**
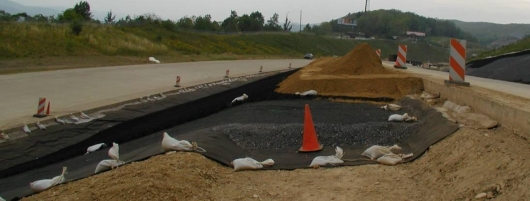
Photo showing cross section of geogrid anchor system.

**Figure 14.**
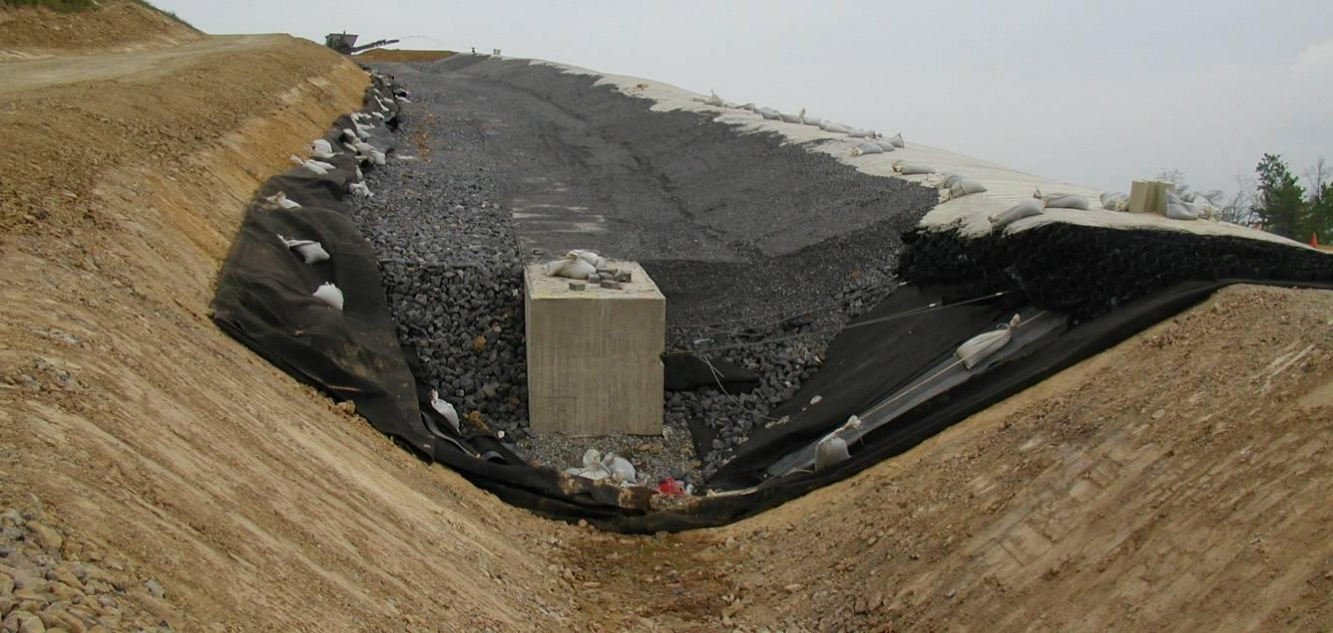
Photo showing cross section of concrete deadman anchor system.

**Figure 15.**
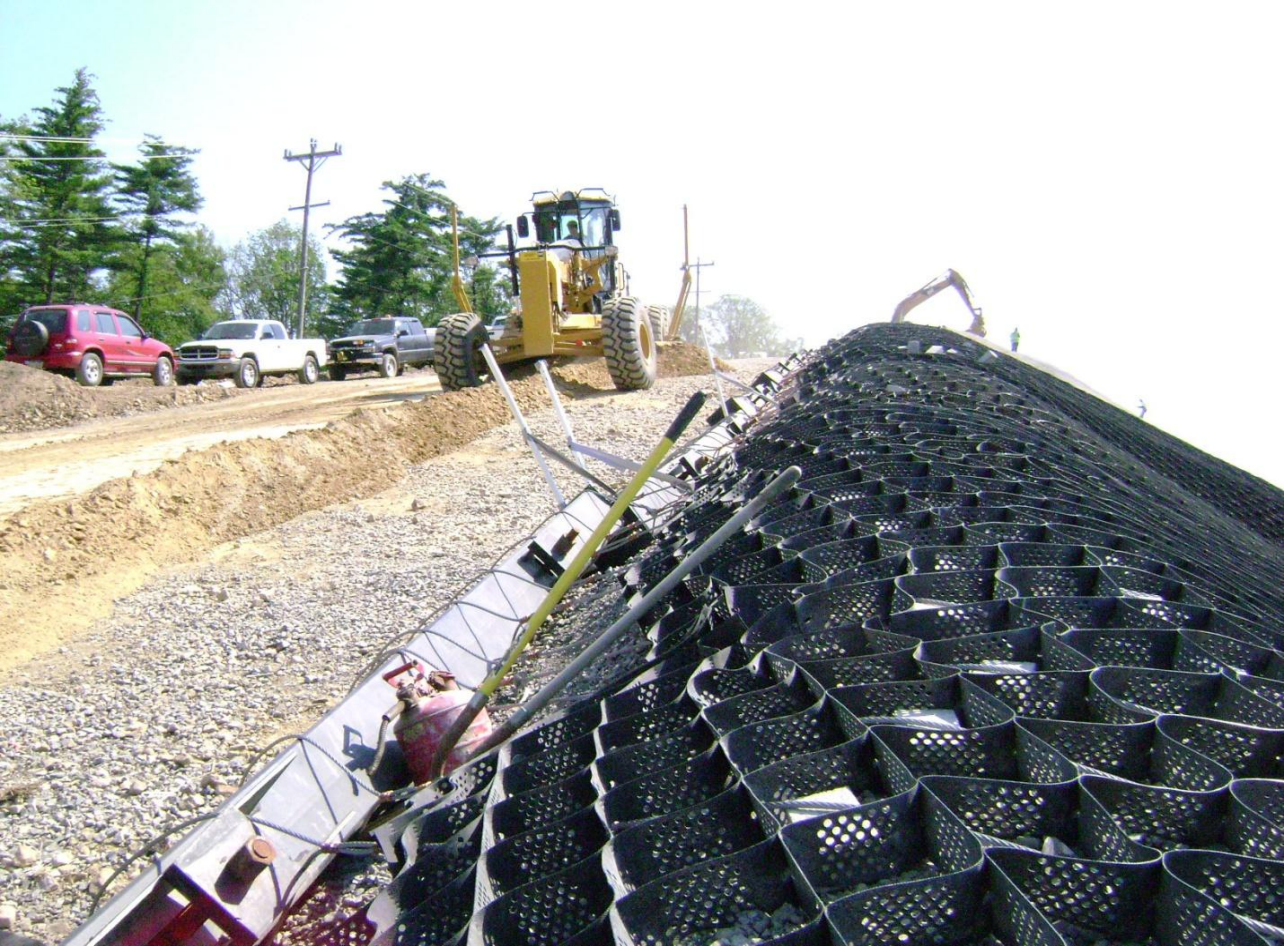
Typical view of anchor beam installation. Tendon-supported geocell panels are visible to the right. Hollow-section structural steel beams with ground anchor rods and bearing plates are visible to the left.

Load from the armor support systems described in the previous section needs to be transferred to the ground in order to support the cover. This load transfer is accomplished with a variety of anchors located at the crest of slope. All anchors were located at the crest of slope because numerous penetrations through the geomembrane of the cover would have compromised the barrier function and were therefore not allowed. For the geogrid reinforced sections, the geogrid was anchored through burial in a trench, as shown in Figure 13. Tendon anchors were of two types: (1) concrete deadman or (2) anchor beams. The type of anchor selected for each section depended on the load demand and the available space. The concrete deadman anchor was employed along slopes which had relatively short slopes and abundant space for an anchor. Figure 14 shows a typical deadman anchor for this project. Anchor beams were used everywhere else. Support of the anchor beams was achieved in most cases through the installation of ground anchors. In one exception, where there was insufficient rock available behind the anchor beams to support ground anchor installation, laterally-loaded piles were used to support the anchor beams. A typical view of anchor beam installation is shown in Figure 15.

In addition to the factors of safety mentioned earlier for the armor support reinforcement, safety of the overall system is ensured by sizing each successive element in the load transfer hierarchy to support greater load before failure. Therefore, should an overloading of the cover system occur, any failure would be local and gradual, as opposed to widespread and sudden. For example, the geocell/tendon support network was sized so that overloading of the armor system would result in failure of the tendon/geocell junctions at loads much less than required to break individual tendons. The tendons were sized so that they would break at loads less than required to overstress the anchor beams. The anchor beams were sized to fail at loads less than required to pull out the ground anchors. Accordingly, if the armor system were to be overloaded, possible outcomes include local loss of armor infill, or, in the worst-case scenario, a slow gradual progression of local failures. This design philosophy provides an intrinsic protection against dangerous failures.

### 4.1. Geogrid Anchor

Anchorage of the geogrid reinforcement was achieved by burying the geogrid at the top of slope with sufficient soil to resist sliding by friction. The driving tension force to be resisted by the geogrid anchor was obtained during the sizing and selection of the geogrid in the previous section. Once the allowable tension in the geogrid was obtained, the anchorage at the top of slope was checked to ensure adequate embedment of the geogrid to support this load. These calculations followed a modified version of the equation discussed by Koerner [5]:

(7) $$T_{all}=2C_{i}L_{e}{\sigma^{\prime }}_{n}\text{tan}\phi^{\prime }$$

where *T_all_* is the allowable tension, *C_i_* is a reduction factor accounting for interface friction between geogrid and soil, *L_e_* is the embedment length of the geogrid within the soil anchor, ${\sigma^{\prime }}_{n}$ is the effective normal overburden stress, and φ′ is the effective stress internal friction angle for the anchorage soil. Note that this calculation assumes that the critical interface governing the pullout resistance of the geogrid is the interface on the top and bottom of the geogrid with the surrounding soil. However, in the case of the geogrid anchors, which were located directly above the cover system geosynthetics, the critical interface is the interface at the bottom of the anchor (*i.e.*, a block failure of the soil above the cover, mobilized by the geogrid tension). Therefore, Equation 7 is modified to consider a more relevant sliding block analysis:

(8) $$T_{all}=L_{e}{\sigma^{\prime }}_{n}\text{tan}\delta =L_{e}\gamma_{a}d_{a}\text{tan}\delta$$

where *γ_a_* is the unit weight of the compacted anchor soil and *d_a_* is the depth of the anchor soil. All soil anchors included robust stone aggregate drainage layers to prevent the accumulation of water above the cover within the anchor. For the example geogrid design where *T_all_* = 34.0 kN/m, a minimum anchor length *L_e_* = 4.5 m and anchor depth *d_a_* = 1 m satisfies Equation 8 when *δ* = 22° and *γ_a_* = 18.9 kN/m^3^. Values of anchor length, depth, and unit weight were verified by inspection and testing during anchor construction.

### 4.2. Deadman Anchor

Design of the deadman anchors considered two important design criteria, (1) the anchor must be sized and buried sufficiently to resist the tension load from the loaded tendons at breaking load and (2) the connections from the tendons to the deadman anchor to the ground must be stronger than the tendons. The following subsections describe how the design met these two criteria.

#### 4.2.1. Soil Anchorage Calculation

**Figure 16.**
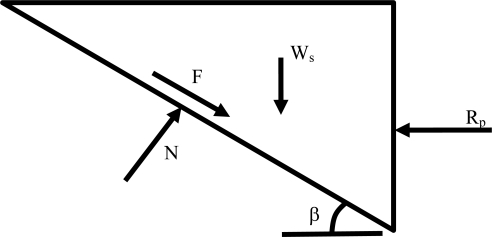
Free body diagram of passive soil wedge against geosynthetics.

The tension in the tendons must be resisted by the weight of the anchor, the frictional resistance at the base of the anchor, and the passive resistance of the soil against the anchor. This resistance can be resolved into horizontal and vertical components. The resulting equations for the factor of safety are therefore:

(8) $$FS_{h}=\frac{R_{f}+R_{p}}{T_{h}}$$

(9) $$FS_{v}=\frac{R_{w}}{T_{v}}$$

where *R_f_* = resisting force due to friction, *R_p_* = resisting force due to the passive soil wedge in front of the anchor, *T_h_* = driving force in horizontal direction, *R_w_* = resisting force due to the anchor’s weight, *T_v_* = driving force in vertical direction. The resisting force due to friction *R_f_* at the anchor’s base is:

(10) $$R_{f}=(W-T_{v})\text{tan}\delta$$

where *W* = weight of the anchor and the soil directly above it, and *δ* = geosynthetic layer interface friction angle (22°). The cover system interface friction angle is used in this calculation because the deadman anchor sits atop the cover system geosynthetics at the crest of slope. Considering only forces acting in the horizontal direction at the face of the deadman anchor (Figure 16), the available resisting force due to the passive soil wedge *R_p_* is:

(11) $$R_{p}=W_{s}\text{cos}\beta (\text{cos}\beta \text{tan}\delta +\text{sin}\beta )$$

where *β* = anchor trench geosynthetic layer slope angle ( = 45°), *W_s_* = weight of soil wedge (1H:1V slope) *=* 0.5*h*^2^*γ_a_*, and *h* = anchor depth.

The cover system interface friction angle is used since the soil wedge in front of the deadman anchor is expected to slide along the weakest interface—the geosynthetic cover system. The vertical component of the tendon tension *T_v_* = (*n*/*w*)*T_ult_*, sin *α*, where *α* = inclination of tendon to the anchor from the horizontal (26.6°). The horizontal component of the tendon tension *T_h_* = (*n*/*w*)*T_ult_*, cos *α*.

Using Equations 8 through 11, the dimensions of each anchor were optimized to yield a factor of safety of at least 1.1 for both the horizontal and vertical components. A relatively low factor of safety is used in this instance since the dead weight of the anchor and backfill soil is verified by testing and numerous resisting forces were ignored. For an example geocell section with two tendons per panel, a 1.2-m wide, 1.2-m tall concrete deadman anchor buried under an additional 1 m of compacted soil yields *FS_h_* = 1.2 and *FS_v_* = 3.1 using these equations.

#### 4.2.2. Load Transfer to Concrete Deadman Anchor

**Figure 17.**
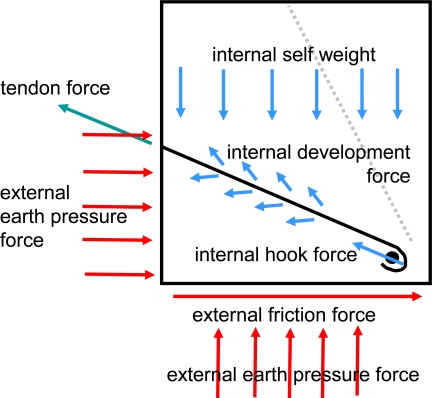
Illustration showing internal and external forces acting on concrete anchor section.

Figure 17 illustrates the internal and external forces acting on the concrete deadman anchor. Load from the tendons is transferred to the ground through the following connections: (1) the tendon is looped through an eyebolt, (2) the eyebolt is threaded into a mechanical coupler, (3) the mechanical coupler is welded to a steel rod, (4) the steel rod is embedded in the concrete anchor, (5) shear and compression forces within the anchor transfer the load to the bottom and front face of the anchor, (6) earth pressure and friction at the anchor faces resist movement of the concrete anchor.

Each of these successive connections in the load path is designed to have a greater reserve of resisting force than the preceding connection. This design approach ensures that potential failures are limited to single connections rather than the entire system according to the discussion at the beginning of this section.

Design of the interior features of the concrete deadman should follow the applicable concrete design codes. In the case of this project, ACI specification 318 [9] was used to check that the steel rod development length, internal shear resistance, and overall steel proportion met code requirements. These calculations verified that adequate development was provided to develop the full tensile strength of the steel rod and that the resulting loads are transmitted through the concrete anchor though compression and shear, ensuring complete transfer of load from the tendons to the ground. For the purposes of these code calculations, the load from the tendons was considered factored load since the breaking of the tendon is a limit state for the system.

### 4.3. Anchor Beam

The purpose of the anchor beam is to transmit loads from the tendons to the ground anchors. The selected structural element for the anchor beam is a rectangular Hollow Structural Steel (HSS) section, also called structural tubing. The ground anchors consist of threaded steel rods secured into the rock with grout and connected to the anchor beam via nuts bearing against steel bearing plates. The ground anchors are installed on the same plan alignment as the tendons which connect to their respective beam. This alignment is maintained to ensure that loads from the tendons are transferred purely in tension to the ground anchors. Because the anchor beam system is passive—loads are only developed in the beam if the tendons are tensioned—production ground anchors were not tensioned during installation.

#### 4.3.1. Beam Sizing

Sizing of the steel components of the anchor beam should follow the applicable steel design code. In the case of this project, the anchor beam design followed the AISC [10] design guidelines for steel members loaded in flexure. The flexural capacity of the anchor beam is evaluated considering that the design tendon breaking force is applied perpendicular to the beam at each tendon location along the beam. This loading is considered factored load in steel design calculations since breaking of the tendons represents a limit state for the system. A loading perpendicular to the beam is conservative since this direction will induce maximum flexural stresses. Finally, due to the connection of two ground anchors to each anchor beam, each anchor beam is modeled as a simply supported beam to calculate design shear forces and moments.

As outlined in AISC [10], an evaluation of the suitability of the selected beam section includes the following design checks: (1) flexure, (2) shear, (3) anchor bearing and (4) tendon bearing. The flexure design checks consider the resistance of the beam section to developing excessive stresses due to bending of the beam. Relevant design checks include gross yielding of the section, local buckling of section components, and lateral-torsional buckling of the section. Relevant shear design checks consider the resistance of the beam section to developing excessive shear stresses due to the applied loads. The anchor bearing checks consider the resistance of the beam section to excessive local deformation due to the action of reactions at the ground anchor contacts bearing plates. Relevant design checks include gross yielding of the web (the two sides of the HSS section) in the area of influence of the ground anchor and web crippling (of a single side of the HSS section) near the ground anchor. Tendon bearing design checks consider the resistance of the beam section to excessive local deformation due to the action of individual tendon loads.

#### 4.3.2. Tendon Slip Restraint Design

Because of the topography of the Large Cut Face, the tendons are oriented at an angle to the perpendicular relative to the anchor beams. Consequently, the tendon load on the anchor beam has both a normal and a tangential component, creating the possibility for the tendons to slip over the beam. To evaluate the possibility of tendon slip, recommended values of the coefficient of interface friction are consulted. According to the bolted connection provisions of ASIC [10], the coefficient of interface friction for steel-to-steel contacts varies between 0.33 and 0.50. This coefficient is therefore conservatively assumed to be 0.33 for design. Accordingly, the maximum angle to the perpendicular that the loaded tendons could assume before the initiation of slip is *δ* = arctan0.33 = 18.3°. Beams installed at angles greater than this value were fabricated with mechanical slip restraints consisting of small steel plates welded to the back faces of the beams. The spacing of these plates matched the spacing of the tendons in each geocell panel. These restraints prevent sliding of the tendon along the anchor beam, ensuring proper load transfer. Most anchor beams did not require tendon slip restraints.

#### 4.3.3. Ground Anchor Bearing Assembly Design

Because of the topography of the Large Cut Face, the tendons are oriented at an angle to the perpendicular relative to the anchor beams. Consequently, the ground anchor tendons must also be installed at an angle to the anchor beam. The ground anchor is supported by a bearing plate which rests on a pair of welded plate supports. The bearing plate supports are sized so that each bearing plate is properly oriented perpendicular to the ground anchor rods when seated on the supports. The bearing plate is checked for bending and shear and the support plates are checked for compression yielding and buckling. The weld between the beam and the support is checked for shearing under the maximum effect of the ground anchor load. For anchor beams supporting the longest slope cover segments, each bearing plate (and hence, ground anchor) is designed to support a 694 kN load. These bearing plates act as beams subjected to central point load and are therefore analyzed for bending. The bearing plates are also checked for shear at their supported edges.

#### 4.3.4. Ground Anchor Design

All tendon load transmitted to the anchor beam is subsequently transmitted to the ground via the ground anchors. The ground anchors consist of threaded steel rods secured into the rock with grout. The design and installation of these ground anchors was performed similarly to retaining wall tiebacks, with the exception that the anchor tendon tension was released following proof testing since the system passively resists loads from the geocell tendons. The ground anchors were designed using the guidelines established in Federal Highway Administration report FHWA-IF-99-015 [11].

The ground anchors are designed to hold an ultimate load of 694 kN. The steel ground anchor tendon rods are 38 mm in diameter and extend to a minimum depth of 12.5 m into the weathered rock near the crest of slope. These anchors were designed with a minimum unbonded length of 3 m and a grouted, bonded length of 9.5 meters.

**Figure 18.**
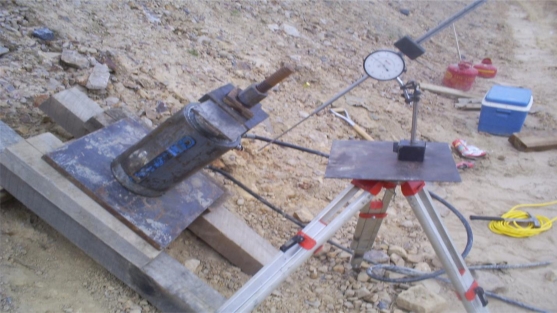
Hydraulic jack being used to proof test a pilot ground anchor. The pictured threaded nut and bearing plate is representative of the nuts and bearing plates used for the production anchors.

The load acting on the anchor is transferred to the rock though the bonded length of the anchor. Prior to load testing the pilot ground anchors, the trial design length of the ground anchors was estimated using the guidelines from FHWA [11]. Using the presumptive ultimate bond strength value of 150 kN/m for weathered rock suggested by [11], the trial design bonded length was specified as 694 kN × 2.0 ÷ 150 kN/m = 9.5 m. Subsequent proof testing (Figure 18) of seven pilot ground anchors verified that the selected bonded length was sufficient to develop the required tension in the ground anchors.

## 5. Conclusions

This paper presented a case history of an armored geomembrane cover design. This cover was installed to prevent the further infiltration of stormwater to pyrite-bearing rocks and thereby prevent further surface and groundwater contamination. The engineering and construction of geomembrane covers for this project differed from typical waste containment practice in that the slopes were steeper than normally encountered and that unreinforced soil cover was not placed above the geomembrane. Additionally, the design requirement of no penetrations through the geomembrane created the need for robust cover anchorage at the crest of slope. The project team elected to use geocells in conjunction with geogrids or steel tendons to support the stone aggregate armor system. The relative novelty of this design approach for steep slopes as long as 110 m allowed the project team to investigate geocell support systems in greater detail.

This paper outlined the various engineering design calculations required to size each component of the cover system to prevent sliding of the armored cover. The overall design goals included providing an overall factor of safety against sliding as well as providing a benign failure mode should some components of the cover system become overloaded. The methodology presented was assembled from several established design practices. This methodology is intended for engineering geomembrane covers on steep slopes, but is applicable to the design of geomembrane covers on both steep and shallow slopes.

Finally, this paper also presented some of the project team’s observations and photographs of the cover system during construction. These observations allowed for comment on the performance of geocell-supported armor systems, comment on the interaction of the friction and tension components of the armor support, presentation of the configuration of practical cover system system anchorage, and project stakeholders’ design concerns. Together with the design philosophy presented, these observations form a record of the project design team’s experience gained during the design and construction of the armored geomembrane cover.

## Figures and Tables

**Table 1.** Cover system interfaces and interface shear strength angle *δ*, adapted from [6].

**Interface**	*δ*
crushed stone-to-nonwoven geotextile	33°
nonwoven geotextile-to-textured HDPE geomembrane	26°
textured HDPE geomembrane-to-nonwoven geotextile	26°
nonwoven geotextile-to-weathered rock	30°

**Table 2.** Cover system design parameters.

**Design Parameter**	**Snow Design Scenario**	**Ice Design Scenario**
Interface Friction Angle	22°	0
Geocell Infill Unit Weight^(1)^	18.1 kN/m^3^	20.4 kN/m^3^
Geocell Thickness	100 mm	100 mm
Overburden Snow Unit Load^(2)^	1.50 kN/m^2^	0

*Notes*:

(1) Ice infill unit weight based on stone porosity = 0.35, ice specific gravity = 0.919, stone specific gravity = 2.70;

(2) Snow unit load based on 610-mm snow cover.

**Table 3.** Summary of geocell laboratory test results performed by GRI.

**Test**	**Reference Standard**	**Average Result**
wide-width tension, break strength (*T_w_*)	ASTM D4885 [7]*	1,030 N
seam peel, seam strength	ASTM D6392 [8]*	1,810 N
washer bearing (2-ply), break strength	n/a—see Figure 8	2,890 N

* Test was performed following procedures similar to the reference standard; test specimens were 100 mm wide.

**Table 4.** Maximum design slope lengths for FS = 1.50 (ice design scenario).

**Slope**	**β**	**Number of Tendons per 2.55-m Wide Geocell Panel**						
		**8**	**7**	**6**	**5**	**4**	**3**	**2**
2H:1V	26.6°	128.7 m	112.5 m	96.6 m	80.5 m	64.3 m	48.2 m	32.3 m
1.5H:1V	33.7°	103.7 m	90.9 m	77.7 m	64.9 m	51.8 m	39.0 m	25.9 m
1.25H:1V	38.7°	92.1 m	80.5 m	69.2 m	57.6 m	46.0 m	34.5 m	23.2 m
1H:1V	45.0°	81.4 m	71.3 m	61.0 m	50.9 m	40.9 m	30.5 m	20.4 m
